# Physiological Rhythms and Biological Variation of Biomolecules: The Road to Personalized Laboratory Medicine

**DOI:** 10.3390/ijms24076275

**Published:** 2023-03-27

**Authors:** Abdurrahman Coskun, Atefeh Zarepour, Ali Zarrabi

**Affiliations:** 1 Department of Medical Biochemistry, School of Medicine, Acibadem Mehmet Ali Aydinlar University, Ataşehir, 34752 Istanbul, Turkey; 2 Acibadem Labmed Clinical Laboratories, Ataşehir, 34752 Istanbul, Turkey; 3 Department of Biomedical Engineering, Faculty of Engineering and Natural Sciences, Istinye University, 34396 Istanbul, Turkey

**Keywords:** biological variation, circadian rhythm, infradian rhythms, physiological rhythms, ultradian rhythms, 3P medicine

## Abstract

The concentration of biomolecules in living systems shows numerous systematic and random variations. Systematic variations can be classified based on the frequency of variations as ultradian (<24 h), circadian (approximately 24 h), and infradian (>24 h), which are partly predictable. Random biological variations are known as between-subject biological variations that are the variations among the set points of an analyte from different individuals and within-subject biological variation, which is the variation of the analyte around individuals’ set points. The random biological variation cannot be predicted but can be estimated using appropriate measurement and statistical procedures. Physiological rhythms and random biological variation of the analytes could be considered the essential elements of predictive, preventive, and particularly personalized laboratory medicine. This systematic review aims to summarize research that have been done about the types of physiological rhythms, biological variations, and their effects on laboratory tests. We have searched the PubMed and Web of Science databases for biological variation and physiological rhythm articles in English without time restrictions with the terms “Biological variation, Within-subject biological variation, Between-subject biological variation, Physiological rhythms, Ultradian rhythms, Circadian rhythm, Infradian rhythms”. It was concluded that, for effective management of predicting, preventing, and personalizing medicine, which is based on the safe and valid interpretation of patients’ laboratory test results, both physiological rhythms and biological variation of the measurands should be considered simultaneously.

## 1. Introduction

Since the beginning of time [1], the universe has been changing. Heraclitus, an ancient Greek philosopher (535-475 BC), strongly emphasized ‘change’ in the universe (universal flux) [2]. He asserted that “Life is Flux” [3], which means that the only constant in life is “change”. To survive in a changing environment, living systems must adapt their internal systems to external cues [4]. This adaptation is observed in all living systems and organized at the cellular level by the molecular clocks; however, the adaptive ability is decreased during aging [4,5,6,7]. Human metabolism is a dynamic process that shows numerous rhythmic or non-rhythmic variations and consequently, variations are inseparable parts of the human metabolism. It should be noted that metabolic variations are not only necessary for the adaptations but they also are required for internal organizations and disease prevention. Moreover, the changes in the amounts of these metabolic variations could be applied for the prediction of disease or the introduction of individual therapeutic methods based on their specific symptoms [8,9,10,11,12,13]. On the other hand, rhythmic or non-rhythmic variations could be applied to 3P (prevention, prediction, and personalized) medicine. 

The variations in the concentrations/activities of molecules observed in individuals can be classified as systematic and random variations. Systematic variations are partly predictable, i.e., in healthy individuals, the time, level, and duration of systematic variations grossly can be predicted before the variation occurs. On the other hand, the time and duration of random biological variations (BV) cannot be predicted as the systematic variations but can be measured using appropriate measurement and statistical procedures. To avoid terminological confusion, in this manuscript, rhythms (or physiological/biological rhythms) and biological variations (BV) are used to denote systematic and random variations, respectively. 

In biological systems, rhythmic variations in the concentrations/activities of molecules can be observed over a wide range of time scales from fractions of a second to years. These variations are classified into three subgroups: ultradian [14], circadian [11], and infradian [15]. On the other hand, random BV can be observed using serial measurements of analytes from samples taken at the same time on different days. For each analyte, two types of random BVs have been determined: between-subject BV (CV_G_), which is the variation among the set points of the analyte from different individuals, and within-subject BV (CV_I_), which is the variation of the analyte around its homeostatic set point. 

This systematic review aims to describe the two main types of variations: biological rhythms and random biological variation. Accordingly, we have summarized some of the research describing the practical use of these variations in personalized clinical and laboratory medicine in the following sections. 

PubMed and Web of Science databases were chosen as the main databases. Both databases were searched for biological variation and physiological rhythm articles in English language without time restrictions with the terms “Biological variation, Within-subject biological variation, Between-subject biological variation, Physiological rhythms, Ultradian rhythms, Circadian rhythm, Infradian rhythms”. The study was carried out between November 2021 and May 2023. Totally, 225 publications including 217 papers, 5 books, 2 websites, and 1 guideline were selected and included in the study. A total of 152 (70.0%) papers were published within the last decade. 

## 2. Biological Rhythms

Biological rhythms are the inherent rhythmicity observed in living systems that are characterized by any behavioral, physiological, or molecular events [16]. Different rhythms have been investigated at the organismic level ranging from milliseconds of a nerve discharge to the annual rhythms of hibernation and even longer [17]. 

Endogenous rhythms are inseparable parts of almost all living systems ranging from photosynthetic prokaryotic cells to higher-level organisms that act as regulators to adapt internal biological processes to external environments [18,19]. In healthy individuals, biological rhythms are interconnected and cooperate like a symphonic orchestra at all levels of the metabolic organization. 

**Figure 1:**
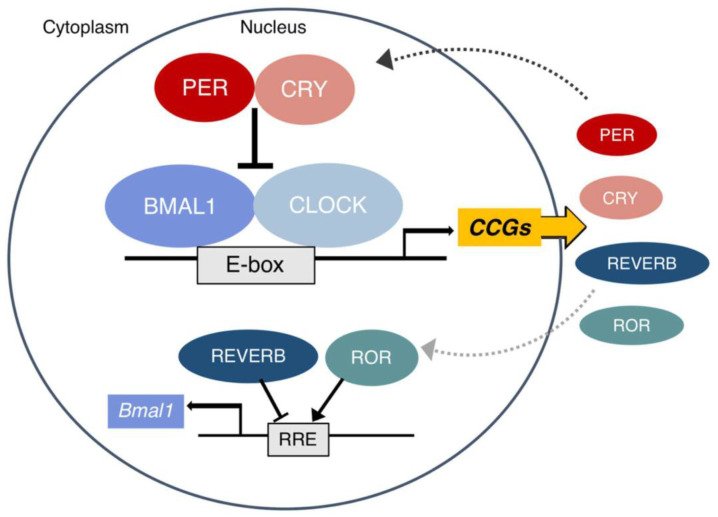
The feedback loops in a molecular clock. The complex CLOCK and BMALI1 rhythmically binds E-box and activate CCGs and other parts of the clock. The activity of CLOCK and BMALI1 is under the control of PER and CRY proteins. They translocate to the nucleus and inhibit CLOCK-BMAL1. The second loop regulates the transcription of Bmal1, which is inhibited by Reverb and activated by ROR. Reprinted from Ref. [52].

Rhythmicity is regulated by biological clocks, which are complex systems consisting of multiple oscillators that each has at least one feedback loop (Figure 1). For instance, controlling the temperature is one of the most important characteristics of biological clocks, so it is critical for a biological system to have a similar oscillation period over a wide range of temperatures [16]. 

Various classification systems have been used for the biological rhythms [20]; among the most important of them is the classification based on the geophysical cycle day and night: ultradian (shorter than 24 h), circadian (almost equal to 24-h daily rhythms), and infradian (longer than 24 h) rhythms [21,22].

### 2.1. Ultradian Rhythms

Ultradian rhythms are defined as all types of ‘short-term rhythms’ with a frequency of fewer than 24 h but commonly with periods in the range of 20 min to 6 h [23,24]. Ultradian rhythms have been detected in all types of living systems ranging from eukaryotic cells [25,26] to mammals [27] and are usually phase-coupled to circadian rhythms in healthy subjects, i.e., the ups and downs of ultradian rhythms appear each day at approximately the same time [17] and play crucial roles in the intracellular coherence [28].

Ultradian rhythms are essential for vital organ functions such as respiration, heart rate, peristaltic activity of the gastrointestinal tract, brain electrical activity [22,29], sperm physiology [30], thermoregulation [31,32,33], rapid eye movement (REM)/non-REM (NREM) sleep cycle, etc. [34]. Additionally, they play crucial roles in cell proliferation and coordination of cellular responses [35]. 

Besides, for the maintenance of life, an ultradian-timekeeping is required to coordinate the biochemical events and regulate the metabolism [36,37] by increasing the efficiency of signal transmission. For example, in comparison to the high level of constant hormone release, the pulsatile release is more efficient for the regulation of metabolism. Regarding the laboratory tests, ultradian rhythms such as episodic hormone secretions [38] govern many endocrine functions. For example, the episodic secretion of insulin from the beta-cell of the pancreas and gonadotropin-releasing hormone from the hypothalamus regulate blood glucose levels and reproductive functions, respectively [38]. 

In addition to hormones, the expression levels of various genes [38,39] and concentrations of analytes such as glucose [40,41] are under the influence of ultradian rhythms. This is especially important in the case of sampling time of the analytes whose measurement results are used for various purposes including diagnosis, screening, and monitoring of the patients and also for the determination of the reference intervals (RIs) and clinical decision limits (CDLs) of the measurands.

As described in the Section 4 and Section 5, ultradian rhythms of the measurands have great influence on the diagnosis, screening, and monitoring of patients and should be considered for the safe and valid interpretation of patients’ laboratory test results. 

### 2.2. Circadian Rhythm

The term ‘circadian rhythm’ (from Latin “circa diem”) for the first time was proposed by Franz Haldberg in 1959 to express the daily oscillations of endogenous biological processes associated with the 24-h rotation cycle of the earth [42]. Circadian rhythm describes the 24-h oscillations of biological processes associated with the dark/light cycle and the earth’s daily rotation [19]. Various external stimuli such as light exposure and food intake play dominant roles in the regulation of circadian rhythms and the dark/light cycle is the main external synchronizer of the circadian rhythm [43]. However, it should be noted that the 24-h oscillation time can be changed slightly using different stimuli [44]. 

Circadian rhythms are developed prenatally and progressive maturation is observed particularly after birth [45]. The pattern of these rhythms can be categorized into two phases: activity and feeding phase and rest and fasting phase. Foods taken in the active phase provide the main substrates such as amino acids, fatty acids, and monosaccharides for energy production and synthesis of carbohydrates, lipids, and proteins. During the resting period, the stored compound are mobilized to sustain the homeostasis of the metabolism [46]. 

External photic and non-photic factors have a great influence on circadian behavior. Photic signals are processed by the eye and transmitted to the hypothalamic suprachiasmatic nucleus (SCN) through the retinohypothalamic tract. Melanopsin plays a crucial role in transmitting the photic signals from the eye to the SCN [47]. The SCN has a complex structure composed of approximately 10,000 neurons, which behaves as a cell-autonomous circadian oscillator, and is accepted as the master internal pacemaker, i.e., the central circadian clock [48]. The SCN has critical properties such as temperature compensation, free-running period under constant conditions, and intrinsic rhythmicity that make it an excellent biological oscillator [16]. 

In addition to the central clock, the presence of peripheral clocks in different organs such as the liver, skeletal muscle, heart, lungs, etc., has been reported [19]. In fact, the biological clock for circadian rhythms is present in all known cells of multicellular organisms and synchronizes the tissues with the external environment via a master circadian pacemaker located in the SCN [49,50]. The activity of peripheral clocks is synchronized by the SCN and both of them regulate the daily rhythmicity of metabolisms [51,52]. In contrast to the SCN, photic signals have little effect on peripheral clocks; however, non-photic signals have profound effects on peripheral clocks [53,54]. Although the master circadian clock in the SCN strongly influences peripheral clocks, there is evidence that peripheral clocks can also influence the master circadian clock [55,56].

The molecular mechanisms behind the circadian clocks were deciphered by Michael Rosbash, Jeffrey Hall, and Michael Young, who won the Nobel Prize in Physiology or Medicine in 2017 [57].

The molecular mechanism of circadian rhythmicity is a complex procedure including transcription, translation, post-transcriptional regulation, and protein–protein interactions [49], which are regulated via both positive and negative feedback loops [58,59,60]. The feedback loops in circadian rhythms on the molecular level are summarized in Figure 1. Briefly, it is based on the complex autoregulatory transcription–translation feedback loops that control the rhythmic expression of clock-controlled genes (CCGs), leading to oscillations in numerous molecules and cellular functions. As shown in Figure 1, the complex CLOCK and BMALI1 rhythmically bind E-box and activate CCGs and other parts of the clock. On the other hand, the activity of CLOCK and BMALI1 is under the control of PER and CRY proteins. They translocate to the nucleus and inhibit CLOCK-BMAL1. These proteins are degraded within the cell and a new cycle begins every 24 h. In addition to this loop, there is a second loop that regulates the transcription of Bmal1 (Figure 1). It is inhibited by Reverb and activated by ROR. The detailed feedback loops of the molecular clock can be found in [52,61,62]. 

Although circadian rhythms are based on transcription–translation feedback loops, i.e., clock proteins regulate their own transcription by a negative feedback mechanism, which produce a rhythmic clock gene expression [63], it has been shown that transcription–translation is not a prerequisite for circadian oscillations [64,65,66,67]. For instance, O’Neill et al. [66] demonstrated that in red blood cell peroxiredoxins, a highly conserved family of antioxidant enzymes that play a dominant role in regulating the intracellular peroxide levels [68,69], undergo the 24-h redox cycles, i.e., the nucleus and consequently transcription–translation are not always required for circadian rhythm in humans. 

The change in levels of numerous molecules within 24 h can be analyzed under the big umbrella, circadiomic [22,70]. The detailed molecular mechanism of the circadian rhythm can be found in [19].

**Figure 2:**
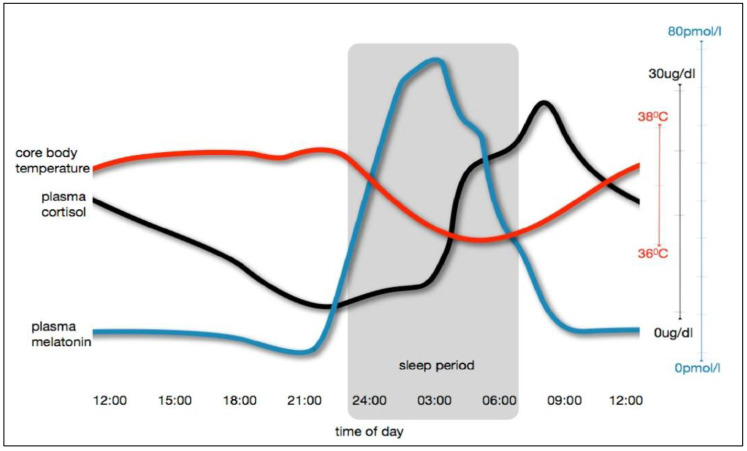
Within-day variations observed in the body temperature and melatonin and cortisol concentrations; reprinted from Ref. [84]. The within-day variation in body temperature is in the opposite direction to the variation observed in melatonin and cortisol.

Circadian rhythms have been observed in numerous laboratory tests including hormones [71] and various analytes such as leukocytes [72,73,74,75], electrolytes [76], trace elements [77,78], glucose [79], etc. Among the hormones, especially melatonin (*N*-acetyl-5-methoxytryptamine) and cortisol come to the fore. Melatonin is secreted by the pineal gland and liver [80] and has strong antioxidant activity and regulates the circadian rhythms of various physiological activities. Its biosynthesis increases at night [81] and is inhibited during daytime by the light detected by the retina [82]. Similar to melatonin, the serum concentrations of cortisol show marked variation within the day with the highest level detected in the early morning [83] (Figure 2). 

### 2.3. Infradian Rhythms

The period of infradian rhythms is longer than the circadian rhythms with ranges from days to years. For biological systems, typical examples are menstruation, hibernation, migration, breeding, molting, etc. [85,86]. In humans, particularly in laboratory medicine, the most important infradian rhythms are observed in analytes that regulate the menstrual cycle and analytes under the influence of sunlight. 

**Figure 3:**
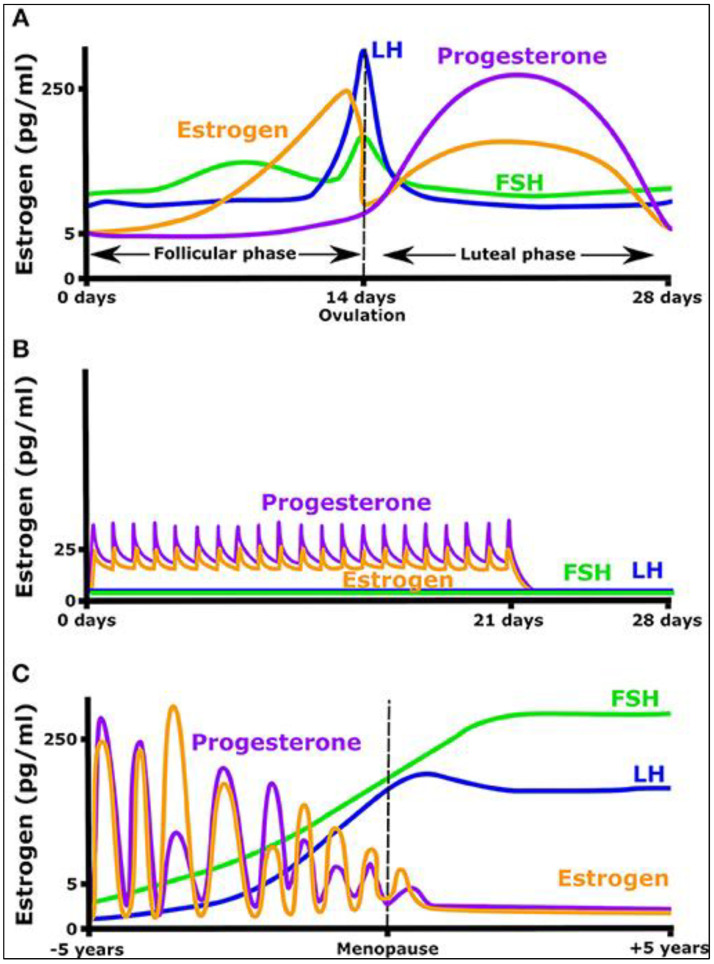
Hormonal changes observed during (**A**) a normal menstrual cycle, (**B**) when taking an oral contraceptive containing both progesterone and estrogen, and (**C**) in the years before and after menopause. Various factors such as pharmacological agents, diseases, or age (a physiological factor) can change the pattern of infradian variations in the analytes; reprinted from Ref. [94].

In the menstrual cycle, pituitary gonadotropins (follicle-stimulating and luteinizing hormones) and ovarian hormones (estrogen and progesterone) show infradian rhythmicity (Figure 3) [87,88]. These hormonal changes that regulate the menstrual cycle in women also have profound effects on other physiological functions, particularly on thermoregulation [89]. In comparison to the follicular phase of menstrual cycle, the core body temperature (CBT) measured in early morning is 0.3 to 0.7 °C higher in the luteal phase [89,90], which is attributed to the thermogenic effect of progesterone [91], and consequently, it can be concluded that in addition to ultradian [31,32,33,92] and circadian rhythms [33,92,93], infradian rhythms is observed in the CBT of women. 

Seasonal infradian rhythms play crucial roles in body functions, particularly in metabolism, reproduction, and immune responses [95]. Recent studies have shown a seasonal variation of immunity and related analytes [95,96,97,98]. Dopico et al. [96] found seasonal expression profiles in more than 4000 protein-coding mRNAs in white blood cells and adipose tissue. They found a profound profile of pro-inflammatory transcriptomic profiles and increased levels of soluble IL-6 receptors and C-reactive proteins during the winter season. Additionally, they found that FcR-gamma-associated processes, B-cell receptor signaling, lysosomes, chemokine signaling, and phagosome were all strongly associated with winter-expressed modules. Pro-inflammatory mediators are associated with various pathological conditions including cardiovascular diseases [99,100,101,102], which is a major cause of mortality and morbidity worldwide [103]. 

Seasonal variation is observed in the analytes which regulate the bone mineral metabolism, particularly vitamin D, which is under the influence of the sunlight. The intensity of sunlight is not globally uniform and shows variations depending on the geographic latitudes and seasons. Depending on the intensity of the sunlight, the levels of 25-hydroxyvitamin D, calcium, and parathyroid hormone (PTH) fluctuate throughout the year. In other words, the concentration of 25-hydroxyvitamin D is higher in the summer and lower in the winter, while the PTH level shows an opposite trend. In the case of calcium, an elevated level is observed in the autumn [104]. 

Additionally, serum/plasma lipid levels also show seasonal variation [105,106,107,108,109,110] between winter and summer. For example, in comparison to summer, serum total cholesterol and LDL-cholesterol levels are increased in winter, but the opposite situation is observed in HDL-cholesterol levels. Detailed information regarding the seasonal variation of the lipids can be found in [111].

Moreover, infradian variations have been observed in various measurands including TSH and thyroid hormones [85,112], sperm physiology [113], urinary excretion of growth hormone [114], enzymes [115,116], etc. 

It should be noted that most of the analytes that are under the influence of infradian variations such as gonadotropins [117,118,119], 25-hydroxyvitamin D, calcium, phosphorus, and parathyroid hormone also show circadian variation [120,121,122,123], as discussed in the following parts.

### 2.4. Interactions among Ultradian, Circadian, and Infradian Rhythms

**Figure 4:**
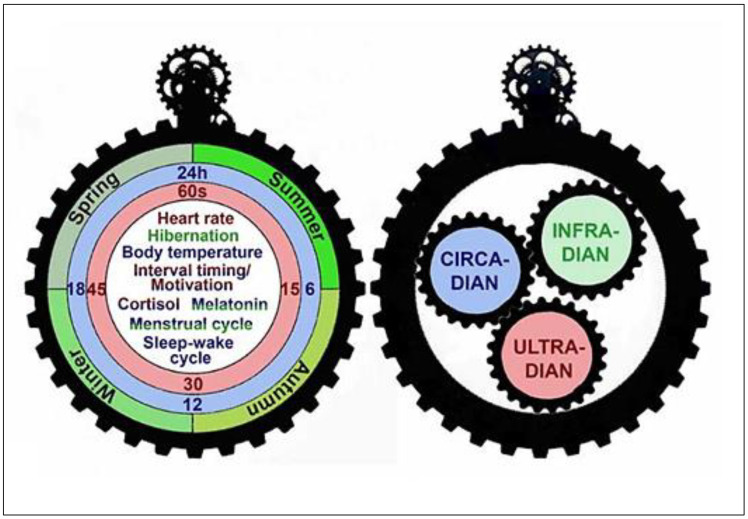
Some metabolic and physiological activities under the influence of ultradian, circadian, and infradian rhythms; reprinted from Ref. [85]. Some analytes and physiological events are under the influence of more than one variation. For example, cortisol secretion has both circadian and ultradian rhythms and the menstrual cycle has infradian and circadian rhythms.

Although it is accepted in theory that biological systems have ‘steady state’ situations and ‘homeostatic set points’ (HSPs) for the measurands, the reality is that the concentration or the activity of biomolecules oscillate as the composite rhythms consist of multiple overlapping oscillations [124] (Figure 4). However, it does not mean that the HSPs of the measurands cannot be determined. In such cases, the HSPs of the measurands can be determined using regression and other trend analysis techniques. 

In comparison to circadian and infradian rhythms, the ultradian events are aperiodic and therefore they are classified as ‘episodic ultradian events’ [24]. Furthermore, while circadian and infradian rhythms are the adaptation mechanisms for predictable environmental changes, at least in theory, ultradian rhythms can be considered as the adaptation mechanisms for the unpredictable environmental changes [24]. It should be noted that the circadian clock behaves as a modulator between ultradian and infradian rhythms [85]. 

### 2.5. Disruption of Biological Rhythms

In comparison to ultradian and infradian rhythms, the disruption of circadian rhythms has been analyzed in detail. Various factors such as shift work [125], jet lag [126], social jetlag [127,128], exposure to artificial light [129], and irregular eating time [130] disrupt the circadian rhythms. Disruption of circadian rhythms lead to numerous serious health problems such as diabetes mellitus [131], atherosclerosis [132], autoimmune diseases [133], obesity [134], cancer [135], insomnia [136], etc. Unlike circadian rhythms, limited data related to the disruption of ultradian and infradian rhythms are available [137,138,139,140].

## 3. Biological Variation

The physiological concentration or activities of biomolecules measured in the same time interval are different in different individuals. The nature of BV is different from that of the physiological rhythms. The BV data of analytes have been derived from the data of the repeated measurements of the analytes [141] rather than the experimental models or animal studies specifically designed for this purpose. For all known analytes that have BV data in the literature, the calculated total variation of repeated measurement results was found to be higher than the analytical variation (the variation of the measurement systems), indicating the presence of additional variation, i.e., biological variation. 

The sampling time for repeated measurements is crucial to obtain reliable BV data. To eliminate the effect of ultradian variation, all samples should be taken at the same time of the day, i.e., measurements of analytes should not be done by combining the samples, some taken in the morning and some in the evening. Similar care should be taken to eliminate the infradian variation of the analytes. Additionally, all samples should be analyzed in a single run to eliminate the between-run analytical variation. 

For an analyte, BV has two main components: between-subject and within-subject biological variations [141]. These two main variations are also the fundamental elements of personalized laboratory medicine. 

### 3.1. Between-Subject Biological Variation

**Figure 5:**
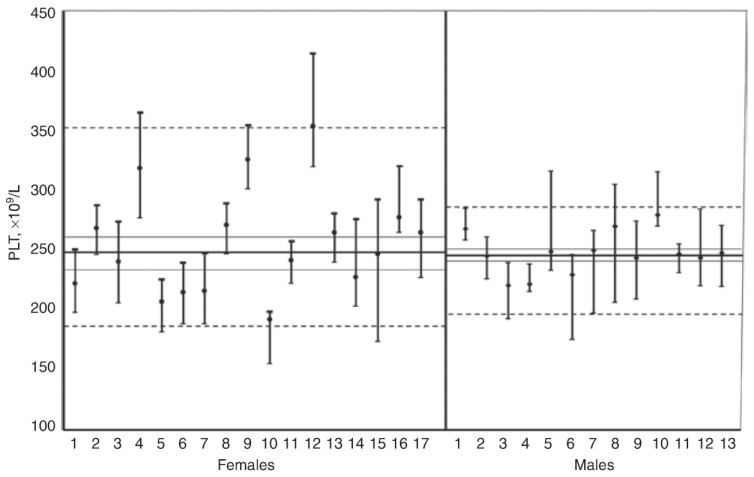
Median values with range (minimum–maximum) of platelet count for individuals based on weekly samplings for 10 weeks. Dashed lines indicate the 5th and 95th percentiles, and the continuous line is the median value with 95% CIs. Reprinted from Ref. [152]. The within-person variation and the set point of each individual are different and additionally, the variation of the set point for males is lower than that for females.

Between-subject BV (CV_G_) is the variation among the individuals’ set points of the analytes (Figure 5). For an analyte, theoretically, it is accepted that each individual has a specific set point, and the concentration of the analytes varies around that set point. It should be noted that the set point of an analyte used to calculate the CV_G_ does not need to be under strict homeostatic control. The set point can be accepted as the mean value of the repeated measurement results of the individual’s data when he/she is at a steady state i.e., the concentration of the analytes is stable. Since the mean value of the analytes is not constant in healthy and diseased individuals and in different age groups, the set point of the analytes can change with age and diseases [141,142]. Depending on the types of analytes the CV_G_ shows a wide distribution. Based on the data given by the European Federation of Clinical Chemistry and Laboratory Medicine (EFLM) Biological Variation (BV) database, it ranges from 1.0% for sodium to 103.4% for cancer antigen 72-4 [143]. In comparison to healthy individuals, currently, there are not sufficient data to illustrate the variation in the set points of the analytes in different clinical situations. 

### 3.2. Within-Subject Biological Variation

Within-subject BV (CV_I_) is accepted as the variation of an analyte around its set point (Figure 5) in an individual. In some analytes such as sodium and calcium, CV_I_ is under strict homeostatic control, while in others, such as serum enzymes, this control is not so strict. The meta-analysis of CV_I_ of more than 200 analytes can be found on the EFLM BV database [143]. Similar to CV_G_, the CV_I_ of analytes shows a wide distribution range from 0.5% for sodium to 135% for adrenalin [143].

The EFLM BV database is a dynamic database, being updated when new data related to the BV of the analytes are available, and therefore the CV_G_ and CV_I_ of analytes may change when the database is updated [143]. 

### 3.3. Within-Person Biological Variation

Although, theoretically, both within-person (CV_P_) and CV_I_ represent the same variation, i.e., the variation of the analytes around the homeostatic set point for an individual, actually they are not exactly the same. The difference between CV_P_ and CV_I_ is the source of the data used to calculate these parameters. The CV_I_ is calculated using the results of the repeated measurements of a group of individuals (population) and therefore it is not specific to the individual, while CV_P_ is obtained using the repeated measurement results of the individual and therefore it is specific to the individual. 

### 3.4. Clinical Applications of Biological Variation Data

Both CV_I_ and CV_G_ are widely used in medical laboratory practice. The BV data have been used: (1) to calculate the index of individuality (II) to evaluate the utility of population-based reference intervals, (2) to calculate the reference change value (RCV), which can be used to make a decision regarding the significance between individual’s serial measurement test results, and (3) to calculate the bias and imprecision to set the analytical performance specification (APS) of the measurement procedure. The details on how to use the BV data for such purposes can be found in [141]. Recently, the CV_I_ and CV_P_ data of the analytes have been used to derive the personalized reference intervals (prRIs) of the analytes [144,145,146,147]. 

Using the prRIs of the analytes may increase the objective interpretation of the test results. As shown in Figure 5, each individual has their own set point and within-person BV. In other words, for an analyte, the variation of the measurands is not limited to a constant set point and a constant variation around the set point. Both the set point and the variation around the set point are individual-specific parameters. Although there are not adequate data reporting the variation of CV_I_ and CV_G_, from the data of the population-based reference intervals of the analytes, it can be speculated that both CV_I_ and CV_G_ change with age and gender [148,149]. For an individual, this makes the variations of the analytes two-dimensional variations. One dimension is the variation of the set point and the other is the variation of CV_I_. To illustrate the variation of the analytes, new studies are required to measure the variation of the analytes at different ages and health statuses.

As the CV_I_ represents the variation in the measurand around its homeostatic set point, although it is not a rule, the CV_I_ of the measurand is expected to increase in pathological conditions related to the measurand. Various studies have reported increasing CV_I_ of specific measurands in individuals with diseases such as diabetes mellitus, chronic kidney diseases, different type of cancers, etc. [150,151].

### 3.5. Reliability of Biological Variation Data

Since the BV data are widely used in the calculation of critical parameters such as RCV, II, APS, and prRI, the reliability of these data is essential [153,154]. The total variation of an analyte can be accepted as the Gaussian combination of pre-analytical, analytical, and biological variation [141]. To obtain reliable BV data from the results of repeated measurements, pre-analytical variation must be minimized; however, it is not so easy in practice and needs strict protocol for pre-analytical procedures and robust statistical techniques [155]. Recently, the EFLM biological variation working group (BV-WG) set up the European Biological Variation Study (EuBIVAS) project, a multicenter study that collected samples from five different European countries using a stringent pre-analytical protocol and robust statistical techniques [155] and updated the BV data of numerous analytes [156]. Additionally, the EFLM BV task group developed the Biological Variation Critical Appraisal Checklist (BIVAC) [157] to evaluate the quality of published BV data and select the appropriate papers for meta-analysis of BV data. The meta-analysis of BV data of numerous analytes is given on the EFLM BV database [143]. In comparison to the historical database [158,159], for most of the measurands, lower values in BV data have been observed. 

### 3.6. Biological Variation in Diseases

Disruption of physiological rhythms has been analyzed in detail [19,51,80,130,134,135,136,160] but the same situation is not the case for BVs. The BV data of measurands were mostly obtained from healthy individuals, and in comparison to healthy individuals, limited data are available for the BV of measurands in patients. 

Indeed, the lack of adequate reliable data for the BV of the analytes in patients limits the usefulness of BV data in clinical practice. Patient monitoring is an important step in the evaluation of the effects/side effects of treatments and the prognosis of the diseases. For this purpose, RCV is used to evaluate the significance between consecutive measurement results. It combines the analytical and biological variations of the measurands and gives the uncertainty associated with the results of consecutive measurements. If the difference between two consecutive measurements results is higher than the RCV, it is accepted as significant, otherwise, it is considered insignificant. However, the problem is that the BV component of RCV is obtained from healthy individuals but used to evaluate the significance between the serial measurements of patients’ test results. This is only valid if there is no significant difference between the measurand CV_I_ values of both patients and healthy individuals. 

Although a BV database for patients is not available, the data of a few papers show that the BV of analytes measured in patients’ samples may be different from the BV of the analytes measured in healthy individuals. In this case, it may not be rational to use the BV values derived from the data of healthy individuals to calculate RCV to monitor patients’ tests results. Parrinello et al. [161] reported the CV_I_ of glucose, HbA1c, fructosamine, glycated albumin, and 1,5-anhydroglucitol in both elderly diabetic and non-diabetic individuals and found elevated levels of CV_I_ for all parameters in diabetic subjects. Within these parameters, the CV_I_ (confidence interval, CI 95%) of glucose, 9.6 (7.3–11.8), and 1,5-anhydroglucitol, 5.7 (4.2–7.2), were found to be significantly higher in diabetic patients than the CV_I_ of glucose, 5.3 (4.6–6.0), and 1,5-anhydroglucitol, 2.9 (2.7–3.2), in non-diabetic individuals. Similarly, Rizi et al. [40] measured the CV_I_ of glucose, insulin, total cholesterol, LDL-cholesterol, HDL-cholesterol, and triglyceride in lean insulin-sensitive and obese insulin-resistant individuals and found elevated levels of CV_I_ for all parameters in obese insulin-resistant individuals. However, due to the lack of CIs in the paper, the significance of the difference between the two groups could not be evaluated. 

In addition to the limited number of papers that measured the BV of some analytes in patients, the reliability of these data is questionable. The individuals must be at a steady state and pre-analytical variation should be minimized, and therefore obtaining reliable BV data from patients’ samples is not an easy task. 

## 4. Physiological Rhythms and Reference Intervals

Clinical decision based on laboratory tests is a comparative procedure and physicians need reference data to compare the laboratory test results. In daily practice, physicians usually use RIs or CDLs as the reference data for comparison [162]. Both RIs and CDLs are powerful tools for diagnosis and screening of the disease. RIs of the measurands can be obtained from the data of healthy population (popRI) or individual (prRI) but CDLs are obtained from patients’ data [162]. 

To derive the popRI, briefly, samples are collected from at least 120 reference individuals [163]. The concentration of the measurands is measured and after excluding the lowest and highest 2.5% of the data using appropriate statistical techniques, the remaining central 95% of the data is accepted as the popRI of the analytes. It should be noted that if partitioning is necessary due to the covariates such as age groups, sex, ethnicity, race, etc., then nx120 (n: the number of covariates) of the reference individuals should be recruited to derive the popRI of the measurands [163]. In comparison to popRI, deriving prRI is an easy procedure. It can be derived using three or more repeated measurement results of the analytes and CV_I_ [147]. However, it is recommended to use CV_P_ instead of CV_I_ and in this case, five or more repeated measurements results are sufficient [144]. 

RIs are obtained from the samples taken in the morning period of the day usually between 8:00–11:00 a.m. However, the ultradian variation of the analyst limits the diagnostic power of the RIs derived from the measurement results of samples taken in the morning time. Since the concentration of the analytes is not constant throughout the day, the within-day variation (ultradian) can be observed in many analytes [164,165,166,167]. Therefore, the sampling time is a critical point in the interpretation of the results of laboratory tests. For example, the measurement results of samples taken at midnight should not be compared to the conventional RI. For such comparisons, the RIs derived from the samples taken within the suitable period are required. Unfortunately, such conventional RIs are not commonly available. For sampling time and reliable comparison with conventional RIs, in addition to ultradian, the circadian and infradian rhythmicity of tests should be considered. For circadian rhythms, the time of the minimum and maximum value of the analytes and additionally the amplitude of the variation, which is defined as one-half of the difference between the minimum and maximum values of the analytes, should be considered [168]. Infradian variation should be considered particularly in estimating the RIs of the analytes showing seasonal variation such as vitamin D, calcium, PTH, total cholesterol, LDL-cholesterol, HDL-cholesterol, etc., and monthly variation such as hormones regulating the menstrual cycle. In clinical practice, population-based RIs (popRI) based on monthly variation is available for hormones [169,170,171] but unfortunately, the popRI based on seasonal data are not common, particularly in routine practice. RIs based on seasonal data for both population and individuals may facilitate the safe and valid interpretation of laboratory test results. 

## 5. Physiological Rhythms and Reference Change Value

Although RCV is a powerful tool for monitoring personal serial measurement results [172], its clinical significance has been criticized [173]. Due to ultradian rhythms of the measurands, RCV should not be used to evaluate the significance between consecutive measurement results of samples taken at different times of the day. For reliable comparisons, the sampling time of the consecutive measurement results should be considered. It should be noted that the CV_I_ used to calculate the RCV is obtained from the data of samples usually taken in the morning period of different days and therefore it should be applied to the measurements results obtained from the samples taken in the morning period of different days. Otherwise, circadian and ultradian variations cannot be eliminated and false positive results may be reported. Moreover, in some cases, sampling at the same time for consecutive days may not be sufficient to eliminate variations other than CV_I_. This can be encountered in the calculation of the RCV for the tests which show infradian variations such as gonadotropic and ovarian hormones, lipids, vitamin D, calcium, etc., especially if the time interval between consecutive measurements approximates the infradian periods of the test. 

## 6. Physiological Rhythms and Chronotypes

Chronotype, or diurnal preference, reflects an individual’s preferred time of the day for the sleep/wake or rest–active cycle [174,175]. Different sleep/wake cycle patterns exist in humans and mainly three different chronotypes can be distinguished: morning types (M-types), evening types (E-types), and neither types (N-types). M-types and E-types are also subdivided into moderate and extreme types [176]. N-types have no circadian preference and can be considered as an intermediate type between M and E-types [176,177]. In comparison to M-types, the E-types have less physical activity and more sedentary time [177]. It should be noted that although sleep is an important dimension, chronotype has multiple dimensions and other factors such as environmental and social influences shape the chronotypes of individuals [178]. The individuals’ chronotypes can be determined using a Morningness–Eveningness Questionnaire [179].

The timing of food intake, which varies depending on chronotypes, has a profound effect on the regulation of biological clocks. Inappropriate timing of food intake (for example during the night) can desynchronize biological clocks and cause adverse health outcomes [180]. It has been shown that more evening chronotypes are associated with obesity [181], type 2 diabetes [182], hypertension [182], mental health issues [183], etc. 

The hormonal profiles of M- and E-types are different. For example, the melatonin profile of M-types differs from E-types [176] and therefore melatonin level can be used as a biomarker for chronotypes [184,185]. In comparison to E-types, the onset, acrophase, and offset of the melatonin profiles occur approximately 3 h earlier in M-types and therefore serum melatonin levels measured particularly around 9:00 a.m. can be used as an indicator to differentiate E-types from M-types since M-types have a lower melatonin level at 9:00 than E-types do [185]. 

Differences have been observed between the laboratory test results of different chronotypes. Vera et al. [186] analyzed the lifestyle, chronotypes, and metabolic syndrome and found that in comparison to M-types, triglyceride and insulin levels and Homeostatic Model Assessment—Insulin Resistance (HOMA–IR) were higher but HDL-cholesterol was lower in E-types. On the other hand, Lucasen et al. [187] found elevated adrenocorticotropic hormone (ACTH) and epinephrine levels in E-types compared to M- and I-types but the elevation of other analytes such as glucose, triglyceride, HDL-cholesterol, and LDL-cholesterol were not significantly different. 

In several chronic pathological conditions particularly cancers, sleep–wake or rest–activity rhythm abnormalities, which are associated with chronotypes, are observed [188]. The rest–activity and cortisol circadian rhythms have been associated with the mortality rates of different cancers including renal [189], lung [190], colorectal [191], and breast cancers [192,193]. Furthermore, the hormonal imbalance between leptin (reduced) and ghrelin (elevated) levels observed in chronotypes with sleep-deprived individuals increases caloric intake, decreases energy expenditure, and leads to weight gain and cardiovascular disease [194,195,196,197]. Additionally, chronotherapy, i.e., optimal timing of treatment could increase the drug efficacy and decrease the side effects of chemo and other therapeutic interventions [198]. 

## 7. 3P Medicine and Variations

As mentioned previously, both systematic and random variations (or rhythmic and non-rhythmic variations) could affect the prediction and prevention of disease and find a way for disease treatment based on the characteristics of the patient. They could significantly reduce the duration of treatment, suggest a more effective therapeutic method, and provide a more comfortable situation for the patient. 

Determining the pattern of physiological rhythms or circadian phenotype for each individual is a major challenge in personalized medicine [199]. A detailed questionnaire including environmental factors such as the duration of the light/dark cycle, exposure to artificial light, information about the individual’s daily habits, nutritional status, physical activities, monitoring of physiological parameters such as blood pressure, etc., will increase the efficacy of targeted therapy and facilitate the planning of personalized treatment. Additionally, algorithms capable of integrating circadian phenotype, personalized RIs, and clinical decision limits will enhance the efficacy of chronobiology in 3P medicine. 

A lifestyle that is compatible with physiological rhythms can prevent various diseases including diabetes mellitus, atherosclerosis, autoimmune diseases, obesity, cancer, insomnia, etc. [19].

People are exposed to artificial light from different sources at different degrees and durations, which has a negative impact on circadian rhythms by suppressing melatonin and phase-shifting the biological clock [200]. Consequently, in the past 50 years, the quality and average sleep duration declined, which is harmful to the health of individuals [200].

All cells in the human body have molecular clocks that oscillate regularly by binding transcription factors to various parts of the genome [201]. Transcription factors binding to numerous genes change the level of different protein and metabolite and posttranslational modifications. Therefore, disruption of circadian rhythms has been linked to numerous diseases such as cancers, diabetes mellitus, obesity, metabolic syndrome, etc. 

Chronopharmacology and chronomedicine will increase the therapeutic effect of drugs and treatment procedures for personalized medicine. It has been shown that the target of numerous drugs shows cyclic gene expression [199,202] and for example, the efficacy of some antihypertensive drugs such as angiotensin II receptor antagonists and Ca channel blockers was found to be higher in the evening time [199,203]. 

No two individuals are identical and this is correct for their chronotypes. Environmental factors such as the duration of the light/dark cycle, exercises, and diet can change tissue specific rhythms [199]. The benefit of physical activity [204] and food intake [205] vary according to the day. Chrononutrition has a great influence on the metabolic and endocrine pathways, which regulate the homeostasis of the organism, and additionally microbiota play a critical role in this interaction [205]. Circadian clock manages the link between homeostasis and nutrition. Overnutrition disrupts circadian rhythms and obesity cause remodeling of circadian activity [206]. Targeted therapy to molecules at their peak expression time may increase the efficacy of treatment. For example, Guan et al. [206] have shown that pharmacological targeting of PPAR-α (a regulator of lipid metabolism) at its peak expression time lowered lipid accumulation in the liver effectively. 

The effect on circadian rhythms is not limited to endocrine and metabolic pathways and the diseases linked to these pathways such as diabetes mellitus, obesity, metabolic syndrome, etc. Since each cell has its own molecular clock, the effect of rhythmic expression of genes can be observed in almost all diseases. For instance, it is revealed that the circadian rhythm could have participated in the duration of bone formation in periodicity-regulated orthodontic tooth movement by manipulating the genes related to the circadian rhythm. Indeed, more effective personalized care could be suggested for maxillofacial surgery and other types of plastic surgery via recognizing the specific environmental conditions and individual genetic and circadian rhythms that could play critical roles in these situations by affecting reconstruction of the soft/hard tissue in a more efficient way [207]. Moreover, variants of the circadian gene in cooperation with the sleep pattern have a relationship with the pathology, development, progression, and aggressiveness of different types of cancer [208,209]. Besides, circadian rhythms are useful for the treatment, prediction, and prevention of several physical and mental diseases including cardiovascular disease, myocardial ischemia, neurodegenerative disease, body microbiota, sleep–wake disorder, etc. [210,211,212,213].

In addition to the circadian rhythm, the application of other types of variations in 3P medicine was also confirmed; however, most of the data are related to the circadian rhythms. For example, evidence show the effects of the ultradian rhythms on heart rate variability, gene expression, locomotor activity, and body temperature [214]. Similarly, infradian rhythms have been associated with reproduction, sperm physiology, telomer length, aging, etc., and the coordination of physiological rhythms are essentials for healthy behavior and memory functions [30,215,216]. Seasonal variations have been observed in telomer lengths [217,218,219] and shortening of telomere length is observed in cellular senescence and accepted as a biomarker for aging [220,221]. 

Infradian rhythms were observed in testis function [222], semen quality [223] and some parameters of sperm physiology such as acrosome reaction (a 12 months cycle) and chemotaxis (a 6 month cycle) [30]. The cyclicity of the biological clock of somatic cells is regulated by specific genes [224]. However, the spermatozoans are transcriptionally inactive cells and therefore the presence of some molecules such as melatonin in the seminal plasma may regulate the variations observed in sperm physiology [225].

## 8. Conclusions

Variations are known as one of the inseparable parts of human metabolism that is classified into two main categories: physiological rhythms and biological variations. Physiological rhythms are crucial for the metabolic adaptation of the organism to external changes, and their disruption leads to serious clinical situations such as cancer, neurodegenerative diseases, insomnia, etc. Unlike physiological rhythms, BV is the random variation of the analytes around its set point (CV_I_) and the variation of set points among individuals (CV_G_). The relation between the disruption of BV and disease has not been analyzed in detail yet; however, in some diseases, the elevated level of CV_I_ has been reported. All analytes measured in medical laboratories from patients’ samples are to some extent under the influence of physiological rhythms. Contrary to physiological rhythms, the BV of the analytes and their potential benefits in clinical practice are not adequately known among clinicians; however, the BV data of the measurands have been used in medical laboratory practice to calculate RCV, II, APS, and prRIs of the analytes. Correct estimations of BV data require detailed knowledge of the physiological rhythms of the analytes. Consequently, for the safe and valid interpretation of laboratory test results, both physiological rhythms and BV of the measurands should be considered simultaneously. These data are useful for effective management of predicting, preventing, and personalizing medicine. 

## Data Availability

Not applicable.
